# Occipital Artery Arising from the Anterior Aspect of the Internal Carotid Artery Identified by Three-Dimensional Computed Tomography Angiography

**DOI:** 10.5812/iranjradiol.7809

**Published:** 2012-06-30

**Authors:** Toshinori Iwai, Toshiharu Izumi, Tomio Inoue, Jiro Maegawa, Nobukazu Fuwa, Kenji Mitsudo, Iwai Tohnai

**Affiliations:** 1 Department of Oral and Maxillofacial Surgery, Graduate School of Medicine, University of Yokohama City, Kanagawa, Japan; 2 Department of Radiology, University and Hospital of Yokohama City, Kanagawa, Japan; 3 Department of Plastic and Reconstructive Surgery, University and Hospital of Yokohama City, Kanagawa, Japan; 4 Department of Radiation Oncology, Southern Tohoku Proton Therapy Center, Southern Tohoku Research Institute of Neuroscience, Fukushima, Japan

**Keywords:** Arteries, Internal carotid artery, Tomography, X-Ray Computed

## Abstract

Variation of the branches of the external carotid artery (ECA) is well known, but it is extremely rare for the occipital artery (OA) to arise from the internal carotid artery (ICA). A 87-year-old man was found to have this anatomical variation on the right side by threedimensional computed tomography angiography for vascular mapping of the carotid arteries before superselective intra-arterial catheterization for advanced tongue cancer. Imaging showed the OA arose from the anterior aspect of the right ICA with the origin located 8.8 mm distal from the carotid bifurcation. The inner diameter of the origin of the OA was 2.1 mm and the angle between the OA and the ICA was 62 degrees. It is important to recognize this anatomic variation of the branches of the ECA before head and neck microsurgical reconstruction or superselective intra-arterial chemotherapy for oral cancer.

## 1. Introduction

Anomalies of the carotid arteries are generally asymptomatic and are discovered incidentally. While variations of the branches of the external carotid artery (ECA) are well known, it is very rare for the occipital artery (OA) to arise from the internal carotid artery (ICA) (1-8). Commonly, the origin of the OA arising from the ICA is at the posterior aspect of the ICA (1-8). To our knowledge, there is only one report of OA arising from the anterior aspect of the ICA (8). We report a case of the OA arising from the anterior aspect of the ICA identified by three-dimension al computed tomography (CT) angiography (3D-CTA).

## 2. Case Presentation

**Figure 1:**
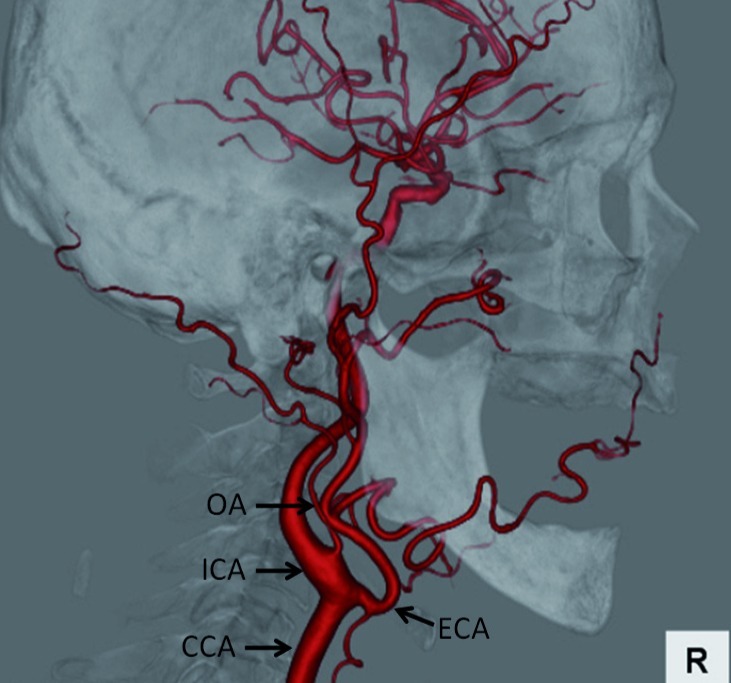
Lateral view of 3D-CTA image of the right carotid artery Abbreviations: ; CCA, Common carotid artery; ECA, External carotid artery; ICA, Internal carotid artery; OA, Occipital artery

An 87-year-old man with left advanced tongue cancer was referred to our department for superselective intraarterial chemoradiotherapy in March, 2011. CT angiography (CTA) was performed for vascular mapping of the carotid arteries before superselective intra-arterial catheterization. A 64-detector spiral CT scanner (Aquilion 64; Toshiba Medical, Tokyo, Japan) was used and nonionic contrast medium (100 mL) was injected at a rate of 4.0 mL/s through an antecubital vein with an automatic power injector. A bolus tracking technique was used to select the individual start delay for the arterial phase. Repetitive low dose scans were performed at a level inferior to the carotid bifurcation with a delay of 8 s. The region of interest was placed in the common carotid artery to measure the bolus arrival time. As soon as an enhancement level of 90 Hounsfield units was reached, the scanning procedure started automatically. Image processing was done on a workstation (Ziostation; Ziosoft, Tokyo, Japan) using the volume rendering technique. Three-dimensional CTA showed the OA arose from the anterior aspect of the right ICA, with the point of origin located 8.8 mm distal from the carotid bifurcation (Figure 1). The inner diameter of origin of the OA was 2.1 mm and the angle between the OA and the ICA was 62 degrees. The patient underwent superselective intra-arterial chemoradiotherapy via the superficial temporal artery (STA) and the OA on the left side and superselective intra-arterial chemotherapy via the right OA arising from the ICA is not necessary for left tongue cancer.

## 3. Discussion

The OA usually arises from the posterior aspect of the ECA opposite the facial artery (3). However, cases of OAs arising from the ICA have been reported as a rare anatomic variation (1-8). Occasionally, the OA arising from the ICA has a common origin with another artery such as the superior thyroid artery or the posterior auricular artery (1, 4). The right OA more commonly arises from the ICA than the left OA according to previous studies (1, 3, 5-7) and the OA arising from the ICA in our case also occurred on the right side. Typically, the origin of the OA arising from the ICA is at the posterior aspect of the ICA (1-8) whereas in our case, the OA arose from the anterior aspect of the ICA. To our knowledge, there is only one report of this variation (8). The OA most frequently arises from the ICA only on one side, but Matsuda et al. (5) reported bilateral OAs arising from the ICAs as a very rare case. Although several authors have reported OAs arising from the ICA 2 cm distal from the carotid bifurcation (3, 5, 6), the OA in the present case originated 8.8 mm distal to the carotid bifurcation.

The embryology of variant origin of the OA remains poorly understood. Lasjaunias et al. (9) hypothesized that the horizontal and distal ascending portion of the OA is a remnant of the proatlantal intersegmental artery. Thus, the OA arising from the ICA is considered to be a persistent proatlantal intersegmental artery and we agree with their hypothesis. An OA arising from the ICA has previously been reported as an anatomic or angiographic finding (1-7). Angiography can show blood flow through carotid arteries, but two-dimensional imaging cannot determine the accurate location of OA origin. Even if an angiographic image shows an OA arising from the posterior aspect of the ICA, the OA may in fact arise from the lateral or medial aspect. In light of this fact, we commonly perform 3D-CTA as it carries no risk of cerebral infarction and can visualize the relationship between the OA and the ECA or ICA from any desired angle. In our case, 3D-CTA as part of the preoperative assessment revealed the OA arising from the anterior aspect of the right ICA.

Superselective intra-arterial chemotherapy is performed via the femoral artery (Seldinger’s method) or the STA (10). For the treatment of advanced oral cancer, we generally perform superselective intra-arterial catheterization via the STA because of long-term catheterization and daily concurrent chemoradiotherapy (10). Retrograde approach via the STA has a lower risk of cerebral infarction than Seldinger’s method which requires several catheter insertions through the carotid bifurcation during the treatment period. However, if the STA is injured intraoperatively or occluded by previous intra-arterial chemotherapy, an approach via another artery such as the OA is necessary to perform retrograde superselective intra-arterial chemotherapy (11). Although the OA commonly arises from the posterior aspect of the ECA opposite the facial artery (3), anatomic variants do occur as decribed above; therefore, it is vital. for the surgical team to radiologically understand the relationships between the OA, lingual artery and facial artery before performing superselective intra-arterial catheterization. In this case, retrograde superselective intra-arterial chemoradiotherapy via the STA and the OA on the left side could fortunately be performed for organ preservation. However, if the OA arises from the ICA on the left side and superselective intra-arterial catheterization was done without preoperative imaging such as 3D-CTA, the catheter inserted into the ICA might cause cerebral infraction. In such case, we should not perform superselective intra-arterial chemotherapy via the OA arising from the ICA, but surgery or superselective intra-arterial chemotherapy via the femoral artery or only the STA to avoid cerebral infarction during interventional radiologic procedures. Three-D-CTA plays an important role in helping the team to determine anatomical variation accurately and surgeons or interventional radiologists should be cognizant that the OA can arise from the ICA as in our case, in order to avoid complications.
